# Quantitative proteomic analysis of extracellular vesicle subgroups isolated by an optimized method combining polymer‐based precipitation and size exclusion chromatography

**DOI:** 10.1002/jev2.12087

**Published:** 2021-04-27

**Authors:** Juan A. Martínez‐Greene, Karina Hernández‐Ortega, Ricardo Quiroz‐Baez, Osbaldo Resendis‐Antonio, Israel Pichardo‐Casas, David A. Sinclair, Bogdan Budnik, Alfredo Hidalgo‐Miranda, Eileen Uribe‐Querol, María del Pilar Ramos‐Godínez, Eduardo Martínez‐Martínez

**Affiliations:** ^1^ Laboratory of Cell Communication & Extracellular Vesicles Instituto Nacional de Medicina Genómica Mexico City Mexico; ^2^ Departamento de Biología Facultad de Química Universidad Nacional Autónoma de México Ciudad de México México; ^3^ Departamento de Investigación Básica Instituto Nacional de Geriatría Mexico City Mexico; ^4^ Human Systems Biology Laboratory Instituto Nacional de Medicina Genómica Mexico City Mexico; ^5^ Coordinación de la Investigación Científica‐Red de Apoyo a la Investigación Universidad Nacional Autónoma de México Mexico City Mexico; ^6^ Department of Genetics Paul F. Glenn Labs for the Biology of Aging Harvard Medical School Boston Massachusetts USA; ^7^ Mass Spectrometry and Proteomics Resource Laboratory Division of Science Harvard University Cambridge Massachusetts USA; ^8^ Laboratorio de Genómica del Cáncer Instituto Nacional de Medicina Genómica Mexico City Mexico; ^9^ Laboratorio de Biología del Desarrollo División de Estudios de Posgrado e Investigación Facultad de Odontología Universidad Nacional Autónoma de México Mexico City Mexico; ^10^ Electron Microscopy Laboratory Instituto Nacional de Cancerología Mexico City Mexico

**Keywords:** breast cancer, exosomes, extracellular vesicles, proteomics, size‐exclusion chromatography, ultracentrifugation

## Abstract

The molecular characterization of extracellular vesicles (EVs) has revealed a great heterogeneity in their composition at a cellular and tissue level. Current isolation methods fail to efficiently separate EV subtypes for proteomic and functional analysis. The aim of this study was to develop a reproducible and scalable isolation workflow to increase the yield and purity of EV preparations. Through a combination of polymer‐based precipitation and size exclusion chromatography (Pre‐SEC), we analyzed two subsets of EVs based on their CD9, CD63 and CD81 content and elution time. EVs were characterized using transmission electron microscopy, nanoparticle tracking analysis, and Western blot assays. To evaluate differences in protein composition between the early‐ and late‐eluting EV fractions, we performed a quantitative proteomic analysis of MDA‐MB‐468‐derived EVs. We identified 286 exclusive proteins in early‐eluting fractions and 148 proteins with a differential concentration between early‐ and late‐eluting fractions. A density gradient analysis further revealed EV heterogeneity within each analyzed subgroup. Through a systems biology approach, we found significant interactions among proteins contained in the EVs which suggest the existence of functional clusters related to specific biological processes. The workflow presented here allows the study of EV subtypes within a single cell type and contributes to standardizing the EV isolation for functional studies.

## 1 INTRODUCTION

In the past few years, the secretion of membrane‐limited vesicles by a variety of cell types has been identified as a novel mechanism of intercellular communication (Pavlyukov et al., 2018; Thomou et al., 2017; Yoshida et al., 2019). These vesicles are actual packages of information that can transfer proteins, nucleic acids, lipids, and small molecules from a parental cell to a recipient cell. Extracellular vesicles (EVs) are originated either from the endosomal pathway (exosomes or small EVs) or the plasma membrane (microvesicles, ectosomes or large EVs) (Mathieu et al., 2019). The current methods available for vesicle isolation (i.e. ultracentrifugation, precipitation, ultrafiltration, and size exclusion chromatography) do not allow the obtention of pure preparations of vesicle subtypes. Despite their different subcellular origin, EVs share some biophysical properties, such as size, density, and some surface markers. Several methodological approaches have been designed to determine the differences in molecular content and biological activities among vesicle subpopulations (Keerthikumar et al., 2015; Kowal et al., 2016; Willms et al., 2016).

A consensus in the field is that EVs are involved in a variety of biological processes both in health and disease. Because the molecular cargo of the EVs reflects the transcriptome and proteome of the cell of origin, there is a growing interest in its characterization to discover new types of diagnostic and prognostic biomarkers (Hurwitz et al., 2016). However, the high complexity of biofluids represents a challenge for isolating EVs and identifying relevant biomarkers of a particular disease. In the case of EV proteome analysis, it has been difficult to determine the exact protein composition because of the co‐isolation of soluble molecules or large protein complexes (Jeppesen et al., 2019). Although ultracentrifugation (UC) is the most common method for EV isolation, the yield of this procedure is considered low and its efficiency is influenced by g‐force, type of rotor, and centrifugation times (Cvjetkovic et al., 2014; Jeppesen et al., 2014). The lack or improper conversion of these parameters is one of the main factors affecting the reproducibility and composition of the sample. Moreover, the sole use of UC does not guarantee a high purity of EV preparations requiring further steps of purification for applications such as mass spectrometry (Takov et al., 2019).

Recently, other less labor‐intensive methods of EV isolation have become popular such as ultrafiltration (UF) and polymer‐based precipitation (Pre). However, the purity in both strategies is compromised due to the co‐isolation of protein aggregates or abundant non‐vesicular proteins (Duong et al., 2019; Nordin et al., 2015; Rider et al., 2016). The advantages of these two methods include the handling of large volume samples from cell cultures (20‐500 ml) and the use of low‐speed centrifugation (Benedikter et al., 2017; Böing et al., 2014; Ludwig et al., 2018). A highly reproducible method to fractionate complex mixtures has been adopted in EV research to reduce the retention of soluble contaminants. In principle, size exclusion chromatography (SEC) separates large particles such as EVs from small particles represented as soluble proteins (Böing et al., 2014). The combination of UC or UF with SEC has been shown to be a successful strategy for mass spectrometry studies and to preserve its biological activity (Takov et al., 2019). A limiting‐step of the SEC method is the need to concentrate the starting sample volume, which could be hundreds of milliliters, to up to 0.5‐1.0 ml when self‐made or commercial columns are used. Commonly, UF is the first step used before loading the sample into the SEC column which can result in an overload of protein, especially when concentrated media comes from volumes larger than 50 ml. Therefore, there is a need to develop a reproducible strategy to simplify EV isolation by SEC, reduce contaminants, and eliminate the dependence on sophisticated equipment.

In this study, we designed a workflow to improve EV isolation based on SEC which is suitable for quantitative proteomics. First, we compared the isolation of EVs‐derived from the breast cancer cell line MDA‐MB‐468 by UC, Pre and SEC in terms of protein yield and abundance of the most common EV markers. Based on the advantages of each method, we found that SEC efficiency improves when the initial step of concentration is performed by the precipitation method. This strategy facilitated the identification of fractions containing EVs revealing putative subtypes of vesicles. Further characterization of the early‐ and late‐eluting fractions, using isobaric labeling for quantitative proteomic analysis, revealed a set of differential enriched proteins in each subgroup. Through a systems biology approach, we identified the main functional modules represented by the common core of proteins found in EVs and got an insight into the functional relevance of their protein cargo for cancer biology.

## 2 MATERIALS AND METHODS

### 2.1 Cell culture and conditioned media collection

Cell lines MDA­MB­468 (ATCC HTB­132) and HEK 293T (ATCC CRL3216), and human gingival fibroblasts were cultured with Dulbecco's Modified Eagle Medium (DMEM) supplemented with 10% (v/v) fetal bovine serum (FBS), 100 U/ml penicillin, and 100 μg/ml‐streptomycin. Primary gingival fibroblasts were obtained from gingival tissue donated by a healthy volunteer at the School of Dentistry of Universidad Nacional Autónoma de México with the corresponding approval of the Ethics Committee. Cells were seeded at a density of 10,000 cell/cm^2^ in 150 cm^2^ flasks and grown at 37°C with 5% CO_2_. For conditioned media collection, cells were grown in DMEM supplemented with 10% of FBS depleted of EVs (Exo‐NM). FBS was ultracentrifuged for 18 h at 118,000 RCF (45Ti rotor, Beckman Coulter) (Shelke et al., 2014). The serum was sterilized by using 0.22 mm filters. The conditioned culture medium was collected after 48 h and centrifuged at 400 RCF for 10 min. (Sorvall Legend RT, Thermo Electron Corporation). Cell viability was measured after each collection, using a TC20 Automated cell counter (Bio‐Rad) and Trypan Blue for cell staining. In all experiments the cell viability was > 96%. Then, the supernatant was centrifuged at 2,000 RCF for 20 min. The resultant supernatant was recovered and stored −80°C prior to further processing. We used equal volumes of pooled media collected from the same cultures to compare the yield of EV recovery from different isolation procedures. For all experiments conditioned media aliquots were thawed on ice.

### 2.2 EV isolation by ultracentrifugation

**FIGURE 1:**
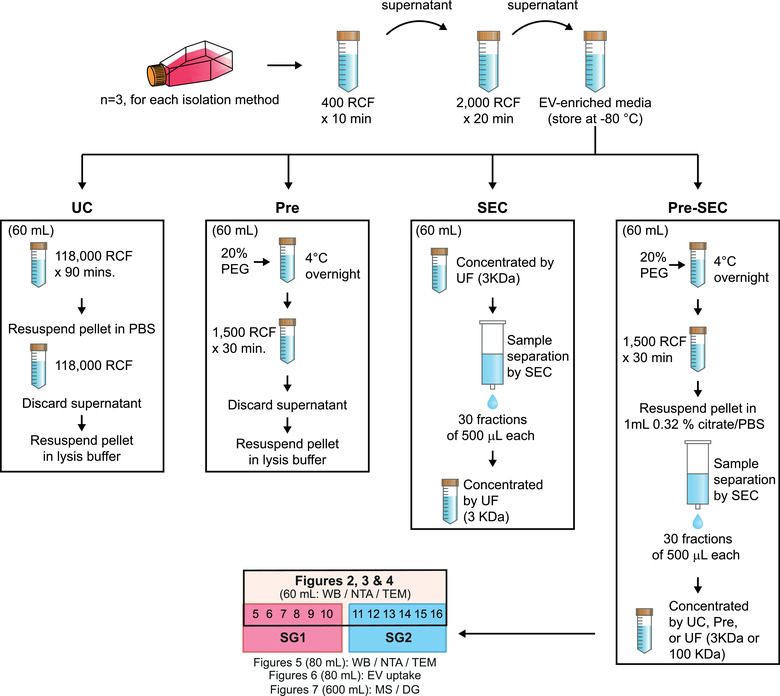
Schematic overview of the EV isolation protocols and experimental workflow. After 48 h, conditioned media was collected and pooled for EV isolation by ultracentrifugation (UC), precipitation (Pre), size exclusion chromatography (SEC), and precipitation coupled to size exclusion chromatography (Pre‐SEC). For further characterization of Pre‐SEC fractions, we separated two subgroups of fractions comprised by fraction 5 to 10 (SG1) and fraction 11 to 16 (SG2). Depending on the downstream analyses the input volume for Pre‐SEC workflow was adjusted as indicated in parenthesis. WB (Western blot); NTA (Nanoparticle tracking analysis); TEM (Transmission electron microscopy); MS (Mass spectrometry); DG (Density gradient)

The conditioned media (60 ml) was transferred into 25 × 89 mm ultracentrifuge tubes (Beckman Coulter) and then loaded into a fixed angle rotor (70Ti rotor, Beckman Coulter) for UC (Optima L‐100 XP, Beckman Coulter) at 118,000 RCF (40,000 RPM, k‐factor 133.4) at 4°C for 90 min (Cvjetkovic et al., 2014). The supernatant was carefully discarded, and the pellet was resuspended in 2.8 ml of PBS. The sample was pipetted into 13 × 51 mm tubes for UC (Optima MAX, Beckman Coulter) at 118,000 RCF (53,000 RPM, k‐factor 54) at 4°C in a fix angle rotor (TLA 100.3, Beckman Coulter) for 39 min. The pellet was resuspended in 110 μl of lysis solution composed of RIPA buffer with 1x protease inhibitors cocktail and 1x EDTA (Halt Protease Inhibitor Single‐Use Cocktail, Thermo Scientific) (Figure 1). The samples were stored at −80°C for further analysis.

### 2.3 EV isolation by precipitation

The conditioned media (60 ml) was supplemented with 50% (w/v) stock solution of polyethylene glycol (PEG) 8000 in 0.5x PBS to a final concentration of 8.3% under sterile conditions. Samples were mixed thoroughly by inversion and incubated at 4°C overnight. The EVs were precipitated at 1500 RCF for 30 min at 4°C. The supernatant was decanted, and samples were spun at 1,500 RCF for 5 min to remove residual media. The pellet was resuspended in 110 μl of lysis solution and stored at −80°C for further analysis (Figure 1).

### 2.4 EV isolation by size exclusion chromatography

We concentrated 60 ml of conditioned media by using an Amicon Ultra‐15 device (Merck‐Millipore) with a 3 kDa cut‐off filter until the volume was reduced to 1 ml. A SEC column was prepared with Sepharose CL‐2B (Sigma‐Aldrich) as described previously (Böing et al., 2014). Briefly, 21 ml of a mixture of 75% Sepharose CL‐2B and 25% PBS containing 0.32% sodium citrate (pH 7.4) was poured into an Econo‐Pac chromatography column (Bio‐Rad). The column was washed with 60 ml of PBS‐citrate to eliminate any ethanol residual from the gel. The resultant column had a diameter of 1.5 cm and a height of 6.2 cm. The sample was loaded on the column and 30 fractions of 500 μl were collected. We measured the absorbance at 280 nm to determine the relative protein content of each fraction with a NanoDrop 2000c (Thermo Scientific). Fractions 5 to 16 were concentrated with an Amicon Ultra‐15 device with a 3 kDa cut‐off filter and stored at −80°C for further analysis (Figure 1).

### 2.5 EV isolation by precipitation coupled to size exclusion chromatography

To increase the purity and yield of EV isolation, we combined the precipitation and SEC protocols (Pre‐SEC) (Figure 1). EVs were precipitated with PEG 8000 (from 60 or 80 ml of conditioned media) and resuspended in 1 ml of PBS‐citrate. Then this sample was fractionated with the Sepharose CL‐2B column as described above. For comparisons with other methods, the fractions containing EVs (5 to 16) were concentrated by UF with a 3 KDa filter Amicon unit. For characterization of early‐ and late‐eluting fractions, we concentrated fractions 5 to 10 as subgroup 1 and fractions 11 to 16 as subgroup 2 according to the following protocols: 1) UC at 118,000 RCF for 39 min (TLA 100.3 rotor), 2) overnight precipitation with PEG 8000, or 3) UF with a 100 KDa filter Amicon unit.

### 2.6 Nanoparticle tracking analysis (NTA)

EVs samples obtained by Pre‐SEC from 80 ml of conditioned media were used to analyze the size distribution and concentration with a NanoSight NS300 (Malvern; software version 3.2.16). The EV fractions were concentrated by UC at 118,000 RCF at 4°C for 39 min. The pellet was resuspended in a total of 400 μl of PBS. Half of this sample (200 μl) was used for NTA and the other half was processed for electron microscopy analysis. The samples were diluted 1:10‐1:1,000 in PBS to assure less than 200 particles per image. The camera level was set at 13 for all recordings. Two 60 s videos were collected for each sample. For analysis, the detection threshold was set at 6. All NTA analyses were performed in triplicate.

### 2.7 Transmission electron microscopy

EVs in PBS obtained by PreSEC (200 μl, see NTA) were mixed with 400 μl of fixative containing 2.5% formaldehyde and 2.5% glutaraldehyde (Electron Microscopy Sciences (EMS)). PBS was exchanged with the fixative solution by using an Amicon‐0.5 ml device with a 3 KDa cut‐off filter and concentrated up to 100 μl. After 45 min of fixation, a 200‐mesh formvar and carbon coated grid (Electron Microscopy Sciences) was placed on top of a 7 μl droplet of EV samples for 20 min. Then, the grid was transferred seven times onto drops of water for 2 min. Negative staining was performed with 4% uranyl acetate for 12 min. Grids were imaged on a JEOL JEM‐1010 electron microscope equipped with an AMT digital camera. Sample preparation and analysis were performed in triplicate.

### 2.8 Gel electrophoresis and Western blot analysis

The Tricine‐SDS‐PAGE method was employed to separate proteins from SEC fractions and vesicle preparations (Schägger, 2006). To visualize proteins, an equal volume of each collected fraction (27 μl) was loaded on a 1.5 mm 10% Tris‐Tricine gel in reducing conditions. Then, the gel was fixed with a solution of 40% methanol and 10% acetic acid for 30 min. After fixation, the gel was incubated with Bio‐Safe Coomassie Stain (Bio‐Rad). In the rest of the experiments, we loaded an equal amount of protein per sample (20 μg) as determined by the BCA assay (Thermo Scientific). For EV marker identification, 10% or 8% gels were used, and proteins were transferred onto a PVDF membrane using a Trans‐Blot Turbo Transfer System (Bio‐Rad). Membranes were incubated overnight with primary antibodies at 4°C in 5% skimmed milk in TBS containing 0.05% Tween‐20 (TBST). We used the following primary antibodies: mouse anti‐ CD81 (Santa Cruz Biotechnology (SCB), sc‐166029) at 1: 500, mouse anti‐CD9 (SCB, sc‐13118) at 1:1,1000, rabbit anti‐ANXA2 (Abcam, ab178677) at 1: 6,000, mouse anti‐ANXA5 (SCB, sc‐74438) at 1: 5,000, mouse anti‐Alix (SCB, sc53540) at 1: 500, mouse anti‐TSG101 (SCB, sc‐7964) at 1: 500, mouse anti‐Fibronectin (SCB, sc‐18825) at 1:5,000, mouse anti‐Flotillin‐1 (SCB, sc‐74566) at 1:2500, mouse anti‐MFG‐E8 (SCB, sc271574) at 1:2,000, mouse anti‐LGALS3BP (SCB, sc‐374541) at 1:5,000, mouse anti‐Rab 5c (SCB, sc‐365667) at 1:2,500, mouse anti‐actinin‐4 (SCB, sc‐393495) at 1:5000, rabbit anti‐albumin (Abcam, ab175934) at 1:100,000, and goat anti‐calnexin (SCBT, sc‐6465) at 1:2,000. Separation of proteins in non‐reducing conditions was performed for the primary antibodies mouse anti‐CD9 (Thermo Fisher Scientific, TS9) at 1:5,000 and mouse anti‐CD63 (SCB, sc‐5275) at 1:2,500. Afterwards, membranes were rinsed three times in TBST for 5 min followed by 1.5 h incubation with IgGκ‐binding protein‐HRP (SCB, sc516102) at 1:2,500/1:5,000, donkey anti‐rabbit IgG‐HRP (SCB) at 1:20,000/1:200,000 or donkey anti‐goat IgG‐HRP (SCB) at 1:20,000 in 5% milk in TBST. After three rinses in TBST, membranes were incubated with SuperSignal West Femto Maximum Sensitivity Substrate (ThermoFisher Scientific) and bands were visualized using a ChemidocTM MP Imaging System (BioRad). The intensity of the bands was quantified using the ImageLab software (BioRad, version 6.0.0 build 25). The relative intensity of each marker was calculated in relation to the intensity of the albumin signal of Exo‐NM processed by Pre‐SEC. All experiments and quantifications were conducted in triplicate for statistical significance.

### 2.9 Transduction of MDA‐MB‐468 cells

For fluorescent protein labeling of EVs, we generated lentiviral particles with the vector CSCW2‐PalmGFP kindly provided by Dr. Xandra O. Breakefield (Massachusetts General Hospital & Harvard Medical School) (Lai et al., 2015). This vector was cotransfected with the plasmids pMD2.g (Addgene plasmid 12259) and psPAX2 (Addgene plasmid 12260) into HEK293T cells using the PureFection Transfection Reagent (System Biosciences). Lentiviral particles were recovered by precipitation with PEG 8000 and used to transduce MDA‐MB‐468 cells using the TransDux Max (System Biosciences), according to the manufacturer's instructions. GFP‐positive (MM468‐PalmGFP) cells were purified on a FACSAria I cell sorter. EVs from the selected cells were isolated according to the Pre‐SEC method.

### 2.10 EV uptake experiments

MDA‐MB‐468 or MCF7 (ATCC HTB‐22) cells were seeded at a density of 20,000 cells/cm^2^ in 8‐well glass slides (Merck‐Millipore). EVs derived from MM468‐PalmGFP or MDA‐MB‐468 cells were isolated by the Pre‐SEC method. The latter EV preparation was labeled with BODIPY TR ceramide according to the manufacturer's recommendations (Thermo Fisher Scientific). Cells were exposed to EVs for 24 h (n = 3). After this incubation, cells were washed twice with DPBS and fixed for 10 min with 3.7% formaldehyde pH7. Cells were counterstained with DAPI and coverslipped with Dako Fluorescence Mounting Medium (Agilent‐Dako). BODIPY experiments were imaged on a Zeiss LSM‐5 Pascal confocal; while GFP experiments were documented on a Zeiss Axio Imager Z2 microscope equipped with an Apotome.2 module. We generated maximum‐intensity projection images using the Zen software (version 3.0.79) from the z‐stack images of the analyzed conditions.

### 2.11 Mass spectrometry analysis

For proteomic analysis, we cultured MDA‐MB‐468 cells in a 5‐layer cell culture flask (NEST Biotechnology Co.) in DMEM supplemented with 1% FBS depleted of EVs at a cell density of 30,000 cells/cm^2^. 600 ml of cell culture media per replicate were processed by the Pre‐SEC method as described above (n = 3). The EV samples were solubilized in truXTRACT buffer DF (Covaris, MA) and subsequently EVs were broken in focused ultrasound Covaris S220 instrument with a 10% abundance over 180 s method. After extraction, proteins were precipitated by 10 volumes of ice‐cold methanol followed by a volume of ice‐cold chloroform. Precipitated proteins were dissolved in 50 μl of 100 mM TEAB (triethyl ammonia bicarbonate) for further digestion. Samples were digested by FASP technology in solution with reduction and alkylation steps. Samples were subjected to TMT labeling and submitted to mass spectrometry analysis without further clean up.

Single LC‐MS/MS experiment that was performed on Orbitrap Elite (Thermo, CA) equipped with Aquity Nano HPLC (Waters, MA) Nano HPLC pump. Peptides were separated onto a 150 μm inner diameter microcapillary trapping column packed first with approximately 3 cm of C18 Reprosil resin (5 μm, 100 Å, Dr. Maisch GmbH, Germany) followed by analytical column ∼20 cm of Reprosil resin (1.8 μm, 200 Å, Dr. Maisch GmbH, Germany). Separation was achieved by applying a gradient from 5%–27% ACN in 0.1% formic acid over 90 min at 200 nL min^−1^. Electrospray ionization was enabled by applying a voltage of 2 kV using a home‐made electrode junction at the end of the microcapillary column and sprayed from fused silica pico tips (New Objective). The Elite Orbitrap instrument was operated in a data‐dependent mode for the mass spectrometry methods. The mass spectrometry survey scan was performed in the Orbitrap in the range of 410 –1,800 m/z at a resolution of 12 × 104, followed by the selection of the twenty most intense ions (TOP20) for HCD/CID‐MS2 fragmentation in the Elite Orbitrap using a precursor isolation width window of 2 m/z, AGC setting of 50,000, and a maximum ion accumulation of 200 ms. Singly charged ion species were not subjected to HCD fragmentation. Normalized collision energy was set to 37 V and an activation time of 1 ms. Ions in a 10 ppm m/z window around ions selected for MS2 were excluded from further selection for fragmentation for 60 s. Samples were analyzed by a single 180 min mass spectrometry run.

### 2.12 Protein identification and quantification

Raw data were submitted for analysis in Proteome Discoverer 2.3.0.81 (Thermo Scientific) software. Assignment of MS/MS spectra was performed using the Sequest HT algorithm by searching the data against a protein sequence database including all entries from human databases and other known contaminants such as human keratins and common lab contaminants. SEQUEST HT searches were performed using a 10 ppm precursor ion tolerance and requiring each peptide N‐/C termini to adhere with Trypsin protease specificity, while allowing up to two missed cleavages. 6‐plex TMT tags on peptide N termini and lysine residues (+229.162932 Da) were set as static modifications while methionine oxidation (+15.99492 Da) was set as variable modification. A MS2 spectra assignment false discovery rate (FDR) of 1% on protein level was achieved by applying the target‐decoy database search. Filtering was performed using a Percolator (64bit version (Kall et al., 2008)). For quantification, a 0.02 m/z window centred on the theoretical m/z value of each of the six reporter ions and the intensity of the signal closest to the theoretical m/z value was recorded. Reporter ion intensities were exported in the result file of Proteome Discoverer 2.1 search engine as an excel table. The total signal intensity across all peptides quantified was summed for each TMT channel, and all intensity values were adjusted to account for potentially uneven TMT labeling and/or sample handling variance for each labelled channel (Kall et al., 2008).

### 2.13 Differential expression analysis

Differential concentration of vesicle proteins of subgroups 1 (SG1) and 2 (SG2) was identified by selecting those proteins whose concentrations were experimentally obtained over the three replicates. The total number of proteins that fulfilled these criteria was 1400. To identify proteins with a differentiated concentration between SG1 and SG2 vesicles, we applied an unpaired two‐sample *t*‐test in R. Test of normality and variance analysis between samples were accomplished through a Shapiro‐Wilk test (*P*‐value < .01) and a variance F‐test (*P*‐value < 0.01), respectively. Differentiated proteins in both conditions were selected by considering a FDR < 0.05 and a log‐ratio (SG1/SG2) > 1. Under these criteria, we identified a total of 148 proteins with differential concentration between SG1 and SG2 vesicles. All analyses were performed using R (3.5.3).

### 2.14 Protein‐protein network interaction

Identification of protein‐protein interactions (PPI) and network visualization were accomplished with STRING 11.0 and Cytoscape 3.7.1 respectively (Shannon, 2003; Szklarczyk et al., 2019). PPI network was obtained by selecting Experiments as the active interactions source with the highest confidence interaction score (0.9).

### 2.15 Functional enrichment analysis

Functional analysis was accomplished considering all sets of proteins detected in the three replicates. To build functionally PPI networks from the collection of proteins inside vesicles, we performed a functional analysis through the app ClueGo 2.5.5 in Cytoscape (Bindea et al., 2009). Functional analysis was done by selecting reactions in Reactome and Wikipathways as databases. We constrained our analysis to the following parameters: a medium network specificity and a *P*‐value < 0.01. The *P*‐Value was corrected with Bonferroni step down. All proteome profiles and code in R are available at “https://github.com/resendislab/vesicles_proteome/”. For enrichment analysis of subsets of proteins, we used FunRich (3.1.3) (Pathan et al., 2017).

### 2.16 Iodixanol density gradient separation

To further analyze the groups separated with the PreSEC method, the EVs corresponding to the SG1 and SG2 were separated by floatation into a iodixanol density gradient (Kowal et al., 2016). Briefly, EV‐enriched fractions were concentrated by UC and the pellet was resuspended in 2.5 ml of a solution of 30% Optiprep (Alere Technologies), 250 mM sucrose, 20 mM HEPES, and 1 mM EDTA (pH 7.4). This vesicle suspension was covered with 1.3 ml of a 20% and 1.2 ml of a 10% iodixanol solutions. The tubes were centrifuged for 1 h at 4°C at 200,000 RCF (46,000 RPM) in a SW55Ti rotor (deacceleration without brake). Then, 490 μl fractions were collected from the top to the bottom. The fractions were diluted with PBS and centrifuged at 118,000 RCF for 39 min in a TLA 100.3 rotor. Pellets were resuspended in lysis solution and protein content was determined by BCA assay.

### 2.17 Statistical analysis

Data were plotted as mean ± SEM using GraphPad Prism (GraphPad Software; version 8.4.2). Statistical comparisons were performed using one‐way ANOVA followed by Tukey's post‐hoc test. A *P*‐value < 0.05 was considered as statistical significance.

### 2.18 Data availability

We have uploaded and submitted all the data of our experiments to the EV‐TRACK database (EV‐TRACK ID: EV200157) (Van Deun et al., 2017). The mass spectrometry proteomics data have been deposited to the ProteomeXchange Consortium (http://proteomecentral.proteomexchange.org) via the MassIVE partner repository (MSV000087057) with the dataset identifier PXD024773.

## 3 RESULTS

### 3.1 Comparison of EV isolation techniques

**FIGURE 2:**
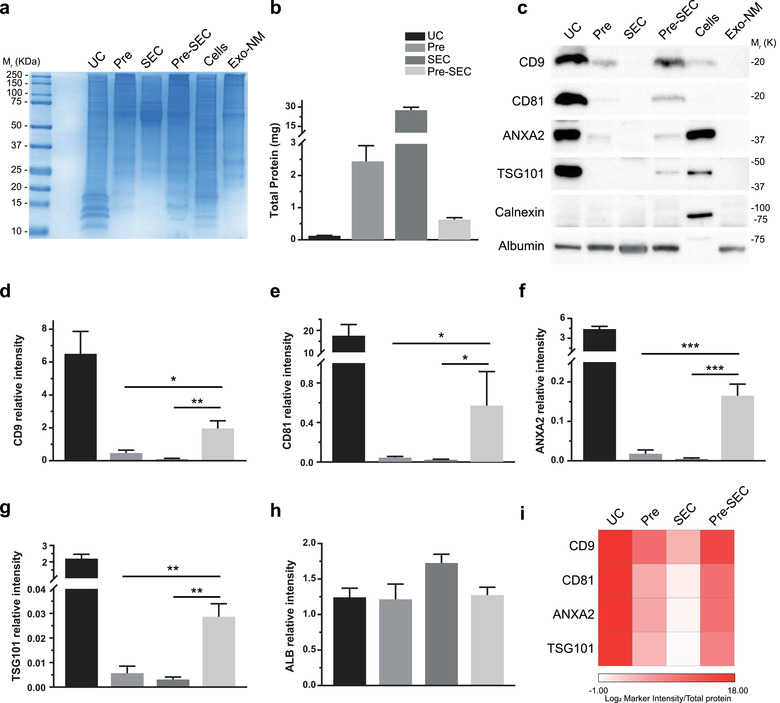
Comparison of isolation methods for EVs. (a) Coomassie blue staining of EV samples obtained by ultracentrifugation (UC), PEG‐based precipitation (Pre), size exclusion chromatography (SEC, concentrated fractions 5–16), and Precipitation‐Size Exclusion Chromatography (Pre‐SEC, concentrated fractions 5–16). MDA‐MB‐468 cell lysate (cells) was used as a positive control and cell culture media prepared with EV‐depleted FBS (Exo‐NM) as a negative control. (b) Total protein obtained from 60 ml of conditioned media (n = 3). Note that the highest recovery was obtained by Pre and SEC. (c) Western blots of EV protein markers detected in samples from different isolation methods at constant protein (20 μg/well, n = 3). Calnexin was used as an intracellular marker of contamination and albumin as an FBS protein contaminant marker. (d‐h) For densitometric analysis, the signal of albumin in Exo‐NM was used to determine the relative intensity of CD9 (d), CD81 (e), ANXA2 (f), TSG101(g) and ALB (h) in samples of different EV isolation methods. (i) EV abundance calculated as the ratio of EV marker intensity to total protein obtained by each method

To determine the yield and purity of the most common techniques for EV isolation, we pooled 240 ml of conditioned media and divided it into four equal aliquots (60 ml). We cultured the breast cancer cell line MDA‐MB‐468 and recovered the conditioned media to measure the abundance of a set of EV markers. A differential protein pattern was observed between UC, Pre, and SEC which revealed an enrichment of proteins below 20 KDa in samples processed by UC (Figure 2a). The amount of recovered protein in the EV fraction also varied drastically depending on the technique employed (Figure 2b). The average yield of protein per milliliter of conditioned media was 2.07 ± 0.18 μg/ml for UC, 40.63 ± 8.14 μg/ml for Pre, and 460.21± 36.35 μg/ml for SEC. The total protein recovered by SEC was two orders of magnitude higher than UC (Figure 2b).

To facilitate the workflow of EV isolation and to increase the yield and purity of the preparation, we precipitated the EVs from conditioned media (60 ml) with PEG and then subjected this pellet to SEC. After concentrating the SEC fractions with an ultrafiltration unit (3 kDa cut‐off), the total protein recovered was five times higher than the protein obtained by UC, with an average yield of 10 ± 0.97 μg/ml of conditioned media (Figure 2b). This strategy, that we denominated as Pre‐SEC, allowed the visualization of EV markers which were undetectable in samples of equivalent amounts of protein of EVs obtained by Pre and SEC alone (Figure 2c). We observed a significant difference in the band intensity of CD9, CD81, annexin A2 (ANXA2), and TSG101 from samples of Pre‐SEC in comparison to Pre and SEC samples. The strongest signal of all the EV markers was obtained by UC (Figure 1d‐1h). The performance of EV recovery of each method was assessed by calculating the EV marker intensity/total protein ratio (Figure 1i). To discard cellular contamination in our preparations, we evaluated the presence of calnexin which was only observable in MDA‐MB‐468 cell lysates. We also corroborated that the cell culture media prepared with FBS depleted of EVs (Exo‐NM) did not show any signal for the evaluated markers. The signal for albumin was similar in all cases.

### 3.2 Characterization of EVs obtained by Pre‐SEC

**FIGURE 3:**
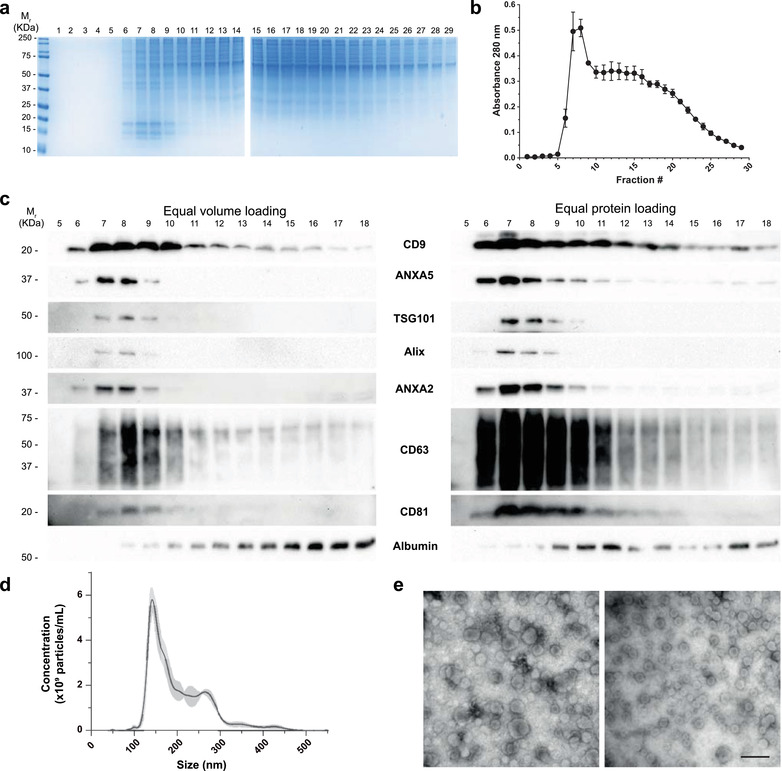
Characterization of MDA‐MB‐468 derived EVs using Pre‐SEC. (a) Coomassie staining showing the protein pattern of 29 fractions collected after Pre‐SEC separation. Notice the enrichment of small molecular weight proteins between fractions 6 to 11. (b) Relative protein content of the 29 fractions collected using Pre‐SEC. The highest values were detected between fractions 6 to 9 (n = 3) (c) Western blots showing the distribution of EV protein markers from fractions 5 to 18. Equal volume (27 μl) or an equal amount of protein (20 μg/lane) was loaded for gel electrophoresis. (d) Nanoparticle tracking analysis of EV samples (n = 3) obtained from fractions 5 to 16 from Pre‐SEC. (e) Transmission electron microscopy of MDA‐MB‐468 derived EVs from fractions 5–16 obtained by Pre‐SEC. Two fields of view are shown to represent the size diversity observed in the sample. Scale bar = 500 nm

Because the combination of methodological approaches enriched the signal of EV markers in the Pre‐SEC preparation, we decided to document the quality and type of EVs obtained by this strategy. We noted that the precipitation, rather than UF (3 kDa unit), of the conditioned media before loading the sample into the SEC column dramatically impacted on the electrophoretic pattern of the proteins as observed by the Coomassie staining (Figure 3a and Supplementary Figure 1a) and the amount of recovered protein (Figure 3b). This analysis revealed an enrichment of a set of protein bands under 20 kDa from fraction 5 to 10 and reduced the amount of protein between 50 and 75 kDa from fraction 9 as compared with SEC preparation (Figure 3a and Supplementary Figure 1a).

To determine the efficiency of the EV isolation among eluted fractions, we performed a Western blot analysis using an equal volume of each fraction. The signal for CD9, annexin (ANXA5), ANXA2, and CD63 was detected starting from fraction 6, while that for TSG101, Alix, and CD81 started from fraction 7 (Figure 3c). Because CD9 and CD63 signal was extended up to fraction 18 and other markers were restricted up to fraction 9 or 10, we loaded an equal amount of protein of each fraction to verify its distribution. Again, TSG101, Alix, ANXA2, ANXA5, and CD81 were mainly distributed from fractions 6 to 11. In contrast the fractions obtained by SEC showed the presence of CD9, ANXA5, ANXA2, and CD63 starting from fraction 7, while TSG101, Alix, and CD81 were not detected (Supplementary Figure 1c). The precipitation of EVs before loading onto Sepharose CL‐2B column also reduced the amount of albumin in EV containing fractions in comparison to the traditional SEC method (Figure 3c‐d and Supplementary Figure 1f ‐g).

To determine the size distribution of the EVs isolated by Pre‐SEC, we pooled the fractions 5 to 16 and performed NTA (Figure 3d) and TEM (Figure 3e). By NTA, we registered that particles had a wide size distribution ranging from 50 nm to 500 nm with a mean size of 204.17 ± 12.33 nm and a mode size of 147.07 ± 5.13 nm. These results were corroborated by TEM analysis where we observed the classical cup‐shaped morphology of EVs. The predominant group of EVs had a size smaller of 200 nm.

**FIGURE 4:**
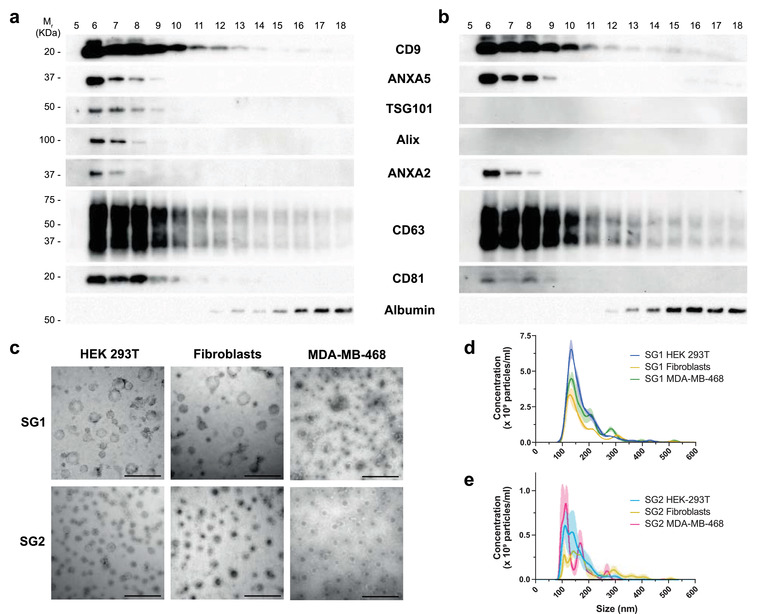
Analysis of EV subgroups obtained by Pre‐SEC of HEK293T, primary fibroblasts, and MBA‐MD‐468 EV samples. Western blot at a constant protein loading (20 μg/lane) showing the distribution of EV markers detected in HEK293T (a) and primary fibroblast (b) EV samples. We divided the fractions in two groups to determine the characteristics of EVs in early‐eluting fractions or subgroup 1 (SG1, fractions 5–10) and late‐eluting fractions or subgroup 2 (SG2, fraction 11–16). (c)Transmission electron microscopy EVs derived from HEK293T cells, primary fibroblasts, and MDA‐MB‐468 cells. We show representative images of each subgroup (n = 3). Scale bar = 500 nm. (d‐e) Nanoparticle tracking analysis (n = 3 for each cell type) of SG1 (d) and SG2 (e) of EVs derived from HEK293T cells, primary fibroblasts, and MDA‐MB‐468 cells

Based on the EV marker signal distribution along the SEC fractions, Pre‐SEC workflow seemed to separate different subgroups of EVs. To evaluate that this separation was not peculiar to MDA‐MB‐468‐derived EVs, we used the same procedure to analyze EVs from HEK‐293T cells and primary fibroblasts. The Western blot analysis of the fractions indicated that the tetraspanins CD9 and CD63 were also distributed up to fraction 18 and markers such as ANXA5, ANXA2, TSG101, Alix, and CD81 were restricted to the early‐eluted fractions (Figure 4a and 4b). In the case of primary fibroblast‐derived EVs, we were not able to detect TSG101 and Alix (Figure 4b). In both cases, the amount of albumin increased in late‐eluting fractions.

To exclude that the signal from tetraspanins in the late fractions would originate from broken EVs, we concentrated fractions 5 to 10 (subgroup 1; SG1) and fractions 11 to 16 (subgroup 2; SG2) and examined these samples by TEM. In all cases we found that both groups contained vesicular structures and there was no obvious evidence of vesicle rupture (Figure 4c). Regarding SG2, there were mostly small vesicles below 200 nm of diameter. The NTA analysis revealed a similar size distribution among the EVs derived from the three cell types analyzed in this work (Figure 4d and 4e). The mean size of SG1 was significantly higher than SG2 in the MDA‐MB‐468‐ (148.9 ± 0.45 nm vs 124.0 ± 0.95 nm; *P* < 0001) and HEK 293T‐derived vesicles (138 ± 0.33 nm vs 129 ± 0.65 nm; *P* < 0.0001). The EVs of the SG2 from the primary fibroblast were bigger than the EVs of the subgroup 1 (149 ± 0.61 nm vs 158.5 ± 1.18 nm; *P* < 0.0001). The particle concentration/total protein ratio is shown in Table 1.

**TABLE 1:** Particles per μg of protein of subgroup 1 (SG1) and subgroup 2 (SG2) of vesicles obtained from MDA‐MB‐468, HEK293T, and fibroblasts cells, using the Pre‐SEC method

Cell line	Particles/mg protein	
	SG1	SG2
MDA‐MB‐468	1.26E+09 ± 8.9E+07	2.37E+08 ± 7.42E+07
HEK293T	9.55E+08 ± 2.25E+08	1.38E+08 ± 1.81E+07
Fibroblasts	7.96E+08 ± 4.60E+08	1.07E+08 ± 6.19E+07

All measurements were performed in triplicate for each subgroup of each cell line. Data intervals represent the SEM.

**FIGURE 5:**
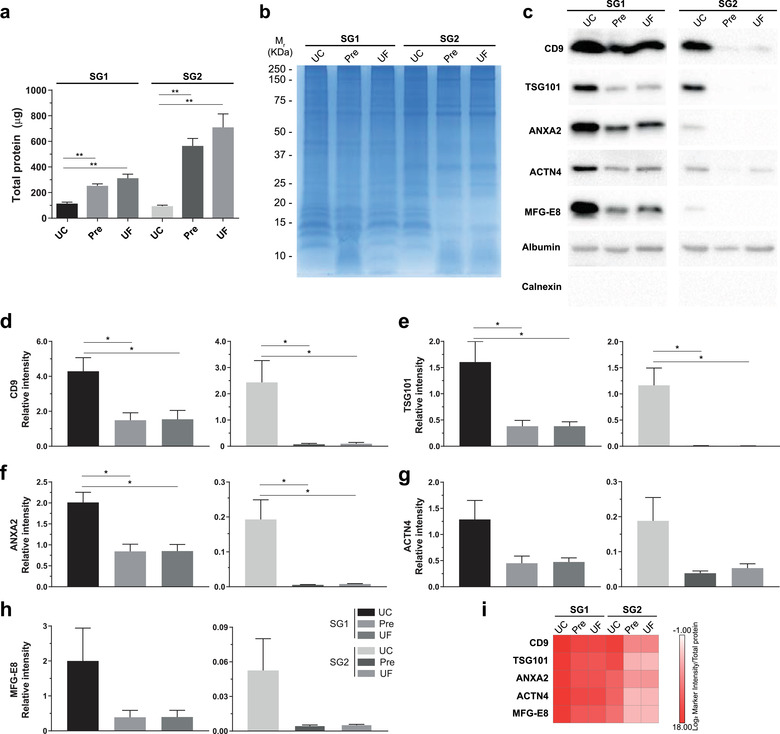
Pre‐SEC yield efficiency after concentration of SG1 and SG2 by different methods. (a) Total protein (μg) obtained from SG1 and SG2 of MDA‐MB‐468‐derived EVs isolated by Pre‐SEC and subsequently concentrated with UC, Pre, or UF. (b) Protein pattern visualized by Coomassie Blue staining from SG1 and SG2 and concentrated by UC, Pre, or UF. (c) Western blot of EV protein markers in samples of SG1 and SG2. (d‐h) Relative quantity (as measured by relative intensity, using Exo‐NM albumin intensity as reference (n = 3)) of EV protein markers CD9 (d), TSG101 (e), ANXA2 (f), ACTN 4 (g), and MFG‐E8 (h) from SG1 and SG2, concentrated by either UC, Pre, or UF. (i) EV abundance calculated as EV marker intensity/total protein ratio

A disadvantage of the SEC procedure is that an EV sample is diluted to 5 or 6 ml which represents an obstacle for downstream applications. We tested among the UC, Pre, and UF (unit of 100 kDa cut‐off) methods which one was the most efficient to concentrate fractions with EVs. In both SG1 and SG2, the highest amount of protein was recovered with the UF method and the lowest with UC (Figure 5a). In SG1 the average yield of protein per milliliter of conditioned media was 1.42 ± 0.15 for UC, 3.16 ± 0.19 for Pre, and 3.90± 0.40 μg/ml for UF. In the case of SG2, the protein yield per milliliter of conditioned media was 1.17 ± 0.09 for UC, 7.06 ± 0.72 for Pre, and 1,764.75 ± 180.93 μg/ml for UF. The electrophoretic protein pattern showed an enrichment of bands below 25 kDa in SG1, independently of the method employed to concentrate the fractions. A similar protein pattern was only observed by UC in the case of the SG2 (Figure 5b). To evaluate the efficiency of EV recovery, we performed a Western blot using antibodies against EV markers shared by several types of EVs (CD9, TSG101, ANXA2) and EV markers enriched in large EVs (actinin A4 (ACTN4) and lactadherin (MFG‐E8)) (Figure 5c). The quantitative analysis showed that the most intense signal of all markers was obtained with UC both in SG1 and SG2. The relative levels of ANXA2, ACTN4 and MFG‐E8 were lower in the SG2, except for CD9 and TSG101 (Figure 5d‐h). The EV marker intensity/total protein ratio showed that Pre and UF showed a low EV recovery for SG2 in comparison to SG1 (Figure 5i).

### 3.3 Uptake of Pre‐SEC isolated EVs by recipient cells

**FIGURE 6:**
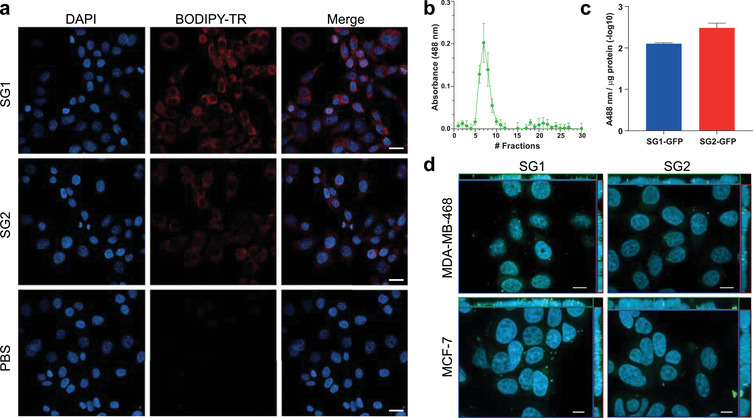
Uptake analysis of EV subgroups. (a) After 24 h of incubation with BODIPY‐TR labelled vesicles, MDA‐MB‐468 cells were imaged in a confocal microscope and a maximum intensity projection image was generated. Almost all cells presented a perinuclear staining. (b) The fluorescence intensity of the 30 fractions collected by the Pre‐SEC isolation method was measured to determine the presence of EVs containing enhanced GFP (n = 3). (c) Relative fluorescence intensity per microgram of protein detected in SG1 and SG2 subpopulations of EVs. (d) Orthogonal projection of optical sections of MDA‐MB‐468 and MCF7 cells incubated with SG1 and SG2 containing enhanced GFP for 24 h. DAPI was used as counterstaining. Scale bar = 10 μm in (a) and 10 μm in (b)

A crucial point of any protocol designed to isolate EVs is to preserve its functional properties. We verified that Pre‐SEC did not affect the capability of EVs to be incorporated in a recipient cell. To this end, we stained the isolated EVs by Pre‐SEC with the lipophilic dye BODIPY‐TR. After 24 h of incubation, most of the MDA‐MB‐468 cells presented a perinuclear staining either with SG1 or SG2 vesicles (Figure 6a). Since lipophilic dyes usually overestimate the degree of EV incorporation, we generated a subclone of MDA‐MB‐468 that releases GFP‐labelled EVs. This strategy allowed us to corroborate whether the GFP fluorescence could be present in the SEC fractions where we previously found the EV markers. The strongest fluorescence signal was observed from fraction 5 to fraction 12 (Figure 6b). After normalizing the fluorescence intensity to the total protein isolated from each subgroup, SG1 and SG2 showed similar levels of fluorescence (Figure 6c). Next, MDA‐MB‐468 or MCF7 cells were incubated with fluorescent vesicles for 24 h. In contrast to BODIPY‐TR, the staining was detected as punctate distributed throughout the cytoplasm in both SG1 and SG2 (Figure 6d).

### 3.4 Differential protein cargo of SG1 and SG2

To determine the protein cargo of EVs isolated by Pre‐SEC, we used TMT‐based isobaric labeling coupled with tandem mass spectrometry. First, we evaluated the impact of different concentrations of FBS in the amount of recovered protein from the Pre‐SEC fractions containing EVs. The precipitation of cell culture media with 10% FBS resulted in an increasing amount of protein from fraction 8, especially above 30 kDa (Supplementary Figure 2a). The reduction of FBS to 1% or its elimination in the cell culture media resulted in a peak of protein from fraction 6 to fraction 14 (Supplementary Figure 2c‐d). The reduction of the FBS concentration to 1% did not alter the pattern of EV markers and produced a clear protein peak where most of the markers were observed (Supplementary Figure 3). Thus, the samples for the proteomic analysis were obtained from cell cultures maintained with 1% of FBS to reduce nonspecific background.

**FIGURE 7:**
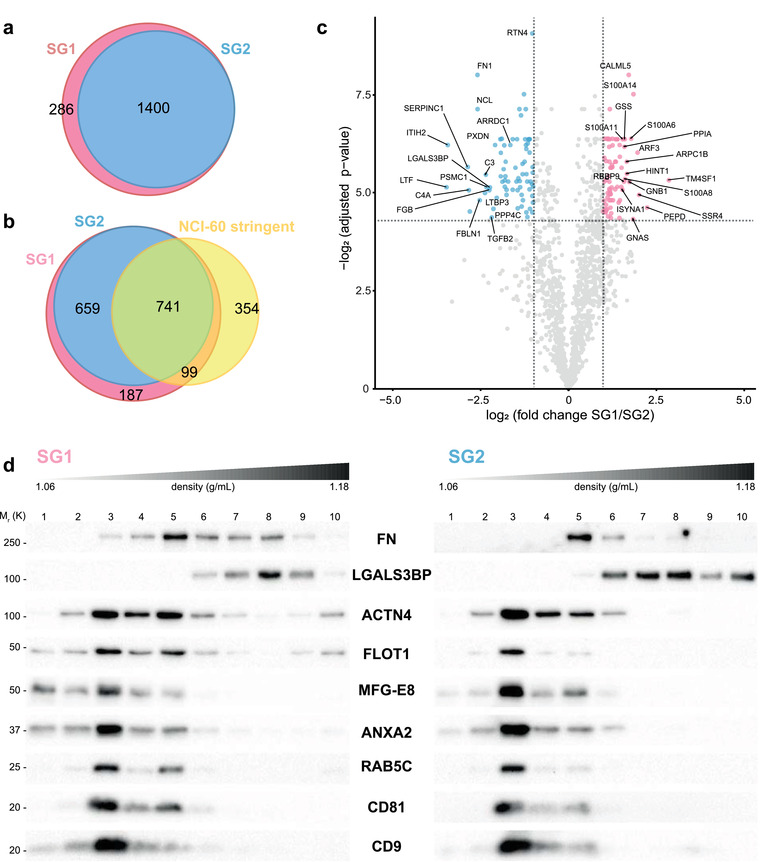
Proteomic analysis of MDA‐MB‐468 EV subgroups. (a) Venn diagram representing the number of proteins identified for SG1 and SG2. (b) Comparison of the number of proteins identified in SG1 and SG2 with a dataset of shared proteins contained in the EVs of 60 cancer cell lines described by Hurwitz et al. (NCI‐60[stringent]). (c) Volcano plot showing the differentially expressed proteins in SG1 and SG2. The significance cut‐off was set to a FDR < 0.05 and absolute log2‐fold change > 1 (n = 3). The red dots represent enriched proteins in SG1 and blue dots represent enriched proteins in SG2. (d) After flotation of SG1 and SG2 vesicles in an iodixanol gradient, ten fractions were collected, and an equal mass of protein was loaded in a Tricine‐SDS‐PAGE. The immunoblots showed that EV markers were in the regions of less density of the gradient. Note that SG1 had enriched labeling of FLOT1, MFG‐E8, ANXA2, and CD9 in fractions 1 and 2

In this study, we identified over 1680 proteins in the three biological replicates of which 1686 were common in all the replicates of SG1 and 1400 in the replicates of SG2 (Supplementary Table 1). Of these proteins, 286 were found exclusively in SG1 (Figure 7a and Supplementary Table 2). Recently, Hurwitz, et. al. have described the EV proteome from 60 cancer cell lines, including that of 6 lines of breast cancer (Hurwitz et al., 2016). A comparison of our protein dataset with a list of proteins shared by at least two‐thirds of the EVs from the 60 cancer cell lines (NCI‐60_stringent_) indicated an overlap of 49.82 % of the proteins (Figure 7b). When the comparison was performed against the specific EV proteome of the MDA‐MB‐468 cell line reported by Hurwitz et al. it resulted in a 75.62% of shared proteins with our data set (Supplementary Figure 4a). Considering both comparisons, a new set of 389 proteins were identified in MDA‐MB‐468‐derived EVs when using Pre‐SEC. A comparison with the Vesiclepedia database showed that 15 of the proteins in our dataset were not previously identified (Supplementary Table 3 and Supplementary Figure 4b).

To further characterize the 286 proteins exclusively found in SG1, we performed a functional enrichment analysis for cellular compartment terms. This analysis showed an enrichment of proteins related to cytoplasm, exosomes, lysosomes, and mitochondria (Supplementary Figure 4c). The most enriched molecular functions included transporter, catalytic and GTPase activity, while the biological processes involved proteins of metabolism, energy pathways and transport (Supplementary Figure 4c and 4d). The core of proteins common to SG1 and SG2 (1400 proteins) had most of its components associated with cytoplasm, exosomes, and lysosomes (Supplementary Figure 4d).

Through a differential expression analysis, we determined the differences in the abundance of the 1400 proteins shared by SG1 and SG2 (FDR < .05 and a log‐ratio (SG1/SG2) > 1). We identified 73 proteins that were significantly up‐regulated in SG1 and 75 significantly up‐regulated in SG2 (Figure 7c and Supplementary Table 4 and 5). The enriched terms in SG1 were associated to signal transduction and cell communication, while SG2 was enriched for cell protein metabolism, and metabolism of nucleotides (Supplementary Figure 5).

To get a better insight into the complexity of SG1 and SG2 and to validate the proteomic data, each subgroup was analyzed using a bottom‐loaded density gradient. This strategy allows to separate soluble proteins from the EVs, being the latter able to float into the regions of less density of the gradient. Interestingly, we were able to observe the presence of putative subtypes of EVs within SG1 and SG2 (Figure 7d). In SG1, we noted higher levels of ANXA2, MFG‐E8, FLOT1, and CD9 in the gradient fractions 1 and 2 as compared with the same fractions of SG2. In the case of SG2, most of the signal of the EV markers was concentrated in fractions 3 and 5. In agreement with mass spectrometry analysis, SG2 showed higher levels of galectin 3 binding protein (LGAL3BP). This protein was in the denser regions of the gradient.

**FIGURE 8:**
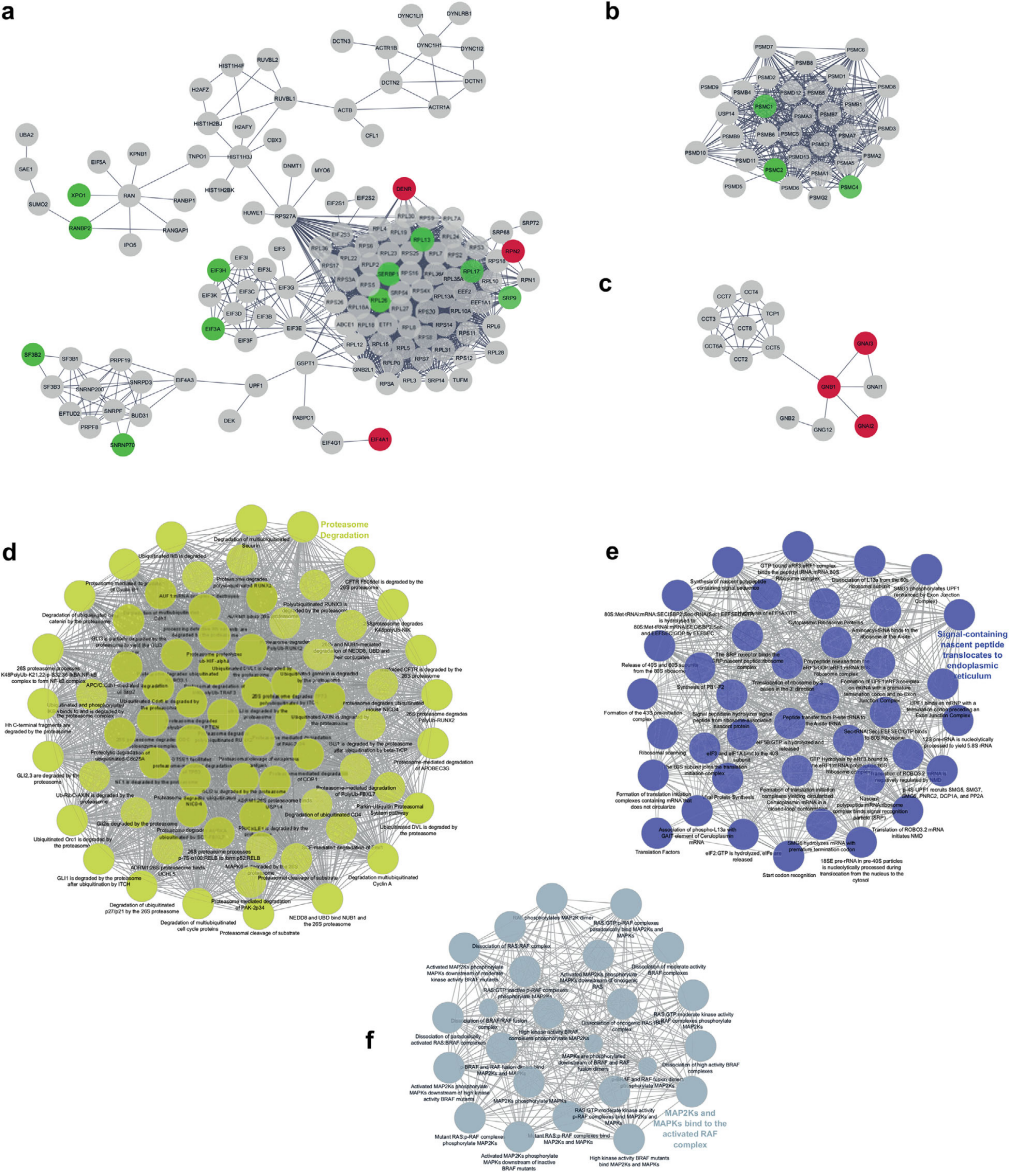
Protein‐protein interaction network of shared proteins by SG1 and SG2. In these subnetworks, the proteins enriched in SG1 are represented as red dots and those enriched in SG2 as green dots. (a) Main subnetwork containing functional modules associated with translation and metabolism of RNA, (b) protein degradation, and (c) protein folding. The main biological processes were related to proteasome degradation (d), translational modulation (e), and MAP2K and MAPK activity (f)​. The network analysis was performed with the STRING software (https://string‐db.org). The network considers reported physical interactions of the 1400 proteins identified in both SG1 and SG2

To have a better understanding of the biology of the EVs derived from the MDA‐MB‐468 cell line, we undertook a systems biology approach by constructing a PPI network from proteome data. Because we did not find exclusive proteins in SG2, the common set of 1400 proteins between SG1 and SG2 was considered as a functional core. The network consisted of 1397 proteins with 2368 interactions (edges). The PPI enrichment p‐value was of < 1.0e^–16^, indicating that the EVs network has more significant interactions than a set of random networks of similar size of proteins drawn from the genome (expected number of edges in random networks:1383). When we exported the network to be visualized in Cytoscape, we identified that it was composed by 417 proteins with at least one interaction, the rest of the proteins were isolated. The proteins presenting interactions were grouped into different subnetworks, of which the one with the highest connections involved clusters of proteins related to translation (ribosomal proteins and transcription factors) and metabolism of RNA (splicing factors and ribonucleoproteins) (Figure 8a). Other relevant subnetworks involved proteins related to protein degradation (proteasome subunits; Figure 8b), protein folding (Figure 8b), cytoskeleton reorganization (Supplementary Figure 6a), tRNA aminoacylation (Supplementary Figure 6b), non‐clathrin‐coated vesicles (Supplementary Figure 6c), clathrin‐coated vesicles (Supplementary Figure 6d), and protein transport (exocyst; Supplementary Figure 6e).

To determine the main biological pathways enriched in the EV proteome of MDA‐MB‐468 cells, we used ClueGo for visualization of the functional clusters. The top significant process were clustered in terms associated with proteasome degradation (pval = 1.37e^–9^; Figure 8d), translational modulation (pval = 1.85e^–29^; Figure 8e), MAP2K and MAPK activity (pval = 3.15e^–6^; Figure 8f), trafficking of clathrin coated vesicles (pval = 4.96e^–10^; Supplementary Figure 7a), chaperonin function (pval = 2.39e^–6^; Supplementary Figure 7b), transport of COPI vesicles (pval = 4.54e^–15^; Supplementary Figure 7c), synthesis of tRNA (pval = 6.41e^–7^; Supplementary Figure 7d), extracellular matrix binding (pval = 1.26e^–7^; Supplementary Figure 7e), metabolic reprogramming (pval = 5.94 e‐9; Supplementary Figure 7f); and formation of spliceosomal complex (pval = 5.72 e^–5^; Supplementary Figure 7 g).

## 4 DISCUSSION

The identification of relevant biomarkers through the molecular characterization of EVs relies on an efficient isolation procedure. Besides the challenge of eliminating protein impurities, there is a need to design strategies that allow the analysis of EV heterogeneity. In this work we compared the performance of the most common isolation methods. Based on the advantages of each method, we designed a workflow that is useful for the fractionation of EV subtypes and suitable for quantitative proteomic analysis. The precipitation of cell culture media before the step of SEC increased the resolution of the traditional SEC method through the elimination of soluble protein. The proteomic analysis and gradient analysis showed that the Pre‐SEC method helps to separate putative EV subtypes secreted by a single cell type.

Alternative methods to UC such as Pre and SEC have been adopted recently due to a higher yield and scalability (Karttunen et al., 2019; Lane et al., 2019; Takov et al., 2019). Here we showed that its sole application does not guarantee the elimination of soluble contaminants. We found that the protein yield was 20 and 220 times higher with Pre and SEC, respectively, than with UC, using the same amount of starting volume of cell culture media. These data indicate that a great proportion of the recovered protein from these methods is not from a vesicular origin, as most of the EV markers were not detected in the Pre and SEC samples. In contrast, the signal was readily observable with the Pre‐SEC strategy. The precipitation step before loading the sample to the chromatography column seemed to eliminate mainly soluble protein and increased the resolution of the SEC method. This is supported by several observations in our study. The electrophoretic pattern of traditional SEC fractions revealed a simple pattern characterized by a great amount of protein which to some extent may correspond to albumin (especially from fraction 11; Supplementary Figure 1). A similar overload of protein has been observed in other studies in which the analysis has been focused on only one or two fractions where the signal of the tetraspanins was detectable (Antounians et al., 2019; Baranyai et al., 2015; Böing et al., 2014; Ludwig et al., 2019). In contrast, we found that CD9 and CD63 were clearly distinguished in over 10 fractions and a clear peak of the material was separated around fractions 5 and 11. Overall, the reduction of protein content in the input sample seems to favor the retention of small vesicles for a longer time in the column. In this regard, the use of crossflow filtration and PEG‐precipitation to reduce protein input for chromatography had a beneficial effect in EV isolation (Mcnamara et al., 2018).

The separation achieved by the Pre‐SEC workflow allowed us to determine the composition of early‐ and late‐eluting fractions containing EVs. Recently, other studies have indicated a great heterogeneity among EVs that is reflected in differences in size, density, and protein and RNA cargo (Gyuris et al., 2019; Jimenez et al., 2019; Kowal et al., 2016; Lai et al., 2016; Willms et al., 2016; Zhang et al., 2018) . Our results indicate that SG1 EVs were larger than SG2 vesicles both in MDA‐MB‐468 and HEK‐293T cell lines. Although there is a size overlap between SG1 and SG2, the Western blot analysis suggested a differential expression of markers depending if the vesicles were contained in the early‐ or late‐eluting fractions. We cannot exclude the possibility that EV subtypes are intermixed due to a delay in migration or an interaction with the SEC column. Our quantitative proteomic analysis revealed a set of proteins that were significantly enriched in each subgroup. The vesicles in SG1 were enriched in ANXA1, ANXA2, and ANXA5. These proteins have been associated with large vesicles derived from the plasma membrane (Jeppesen et al., 2019). Using the iodixanol gradient we noted a type of vesicles in SG1 that floated into the lightest fractions, suggesting that these vesicles are of larger size (Kowal et al., 2016; Willms et al., 2016). Vesicles in SG2 were enriched in molecules including Arrestin Domain‐Containing Protein 1 (ARRDC1), nucleolin, (NCL), and Galectin‐3‐binding protein (LGALS3BP). Interestingly, ARRDC1 and TSG101 are involved in the release of small vesicles formed from plasma membrane (< 200 nm) denominated arrestin domain‐containing protein 1‐mediated microvesicles (ARMMs) (Anand et al., 2018; Jeppesen et al., 2019). In addition, SG2 was enriched in splicing factors and ribosomal proteins and other RNA‐Binding Proteins that have been associated with small vesicles not containing tetraspanins or in some cases with non‐membranous nanoparticles called exomeres (Jeppesen et al., 2019; Zhang et al., 2018). We note that 22% (17/75) of the enriched proteins in SG2 can be contaminants from the FBS which would be expected because in late‐eluting fractions the proportion of soluble proteins should increase. Some of these proteins include lactotransferrin (LTF), inter‐alpha‐trypsin inhibitor heavy chain H2 (ITIH2), antithrombin‐III (SERPINC1), complement C4‐A (C4‐A), and fibulin‐1 (FBLN1) (Shin et al., 2019). Despite these putative impurities, our results suggest that EVs in SG2 constitute a mixture of small vesicles that maintain their functional capabilities as corroborated in the uptake experiments.

The high proteomic similarity between EVs subgroups described here may be due to a technical, rather than a biological aspect. We separated EVs into two subgroups based on properties such as size and elution time through the SEC column. Further fractionation of each subgroup would be necessary to assess whether these subgroups contain true EV subpopulations with different functional roles or even with specific markers. Our density gradient analysis suggested that SG1 and SG2 are comprised of multiple EV subpopulations that may share some molecular markers. Other studies comparing the proteome of large EVs (< 15,000 RFC) and small EVs (> 100,000 RFC) have observed that different subpopulations present between 60% to 96% of common proteins (Jeppesen et al., 2019; Jimenez et al., 2019; Kowal et al., 2016; Willms et al., 2016). A better understanding of EV biogenesis processes is needed to determine the minimum molecular fingerprint representative of different subcellular compartments.

A remaining question in the EV field is about the specificity of sorting mechanisms of proteins and RNA into EVs (Anand et al., 2019). A crucial point for our understanding of EV biology would be the documentation of meaningful interactions among the proteins transported by EVs. Through a systems biology approach, we determined that around 29.8% of the identified proteins can physically interact with at least one other protein present in the EV cargo. The PPI interaction network revealed two modules composed of ribosomal proteins, transcription factors, elongation factors and splicing factors suggesting that MDA‐MB‐468‐derived EVs play a role in modulating protein translation and gene transcription in the recipient cell. Consistent with this observation, it has been reported that glioblastoma‐derived EVs transfer spliceosomal components that are transported to the nuclei of the recipient cell (Pavlyukov et al., 2018). Furthermore, our functional enrichment analysis showed that an important cluster is related to translation regulation indicating a potential of these EVs to modify the cell phenotype locally or at a long distance (Adem et al., 2020). An additional noticeable functional cluster in MDA‐MB‐468‐derived EVs is related to the proteasome complex. Interestingly, triple‐negative breast cancer (TNBC) cell lines, including MDA‐MB‐468, are sensitive to proteasome inhibitors (Milacic et al., 2006; Weyburne et al., 2017). High gene expression levels of immunoproteasome complexes (*PSMB8, PSMB9, and PSMB10*) are related to survival effects in TNBC cell lines (Adwal et al., 2020). The enrichment of this kind of functional modules has been experimentally corroborated in colorectal cancer cells (Choi et al., 2012). The identification of these functional interactions provides information about EV biogenesis and cell phenotype (Choi et al., 2012; Rontogianni et al., 2019). Further studies are required to determine the status of protein networks in non‐pathological conditions.

An objective of dissecting EV heterogeneity is to reveal a molecular signature of specific EV subtypes that provide information about putative biomarkers for diagnosis and prognosis. The Pre‐SEC workflow offers a strategy to separate EVs from a complex mixture based on their time of retention in a SEC column. The implementation of this workflow allowed us to fractionate the EV preparation into subgroups with a distinctive proteomic profile. We note that in SG1 and SG2 EVs there is an enrichment of key molecules that have been previously related to breast cancer at a tumor or cellular level. Among the 10 members of the S100 family in our dataset, S100A14, S100A6, S100A8, S100A11, and S100A16 were up‐regulated in SG1. High levels of these calcium‐binding proteins have been associated with poor prognosis and metastasis of breast cancer (Cancemi et al., 2018; Ehmsen et al., 2015; Zhong et al., 2018). In the case of SG2, the proteins fibronectin and LGALS3BP were enriched and have also been involved in breast cancer progression and considered as prognostic markers (Läubli et al., 2014; Moon et al., 2016; Stampolidis et al., 2015). Our Western blot analysis indicated that these proteins are mainly in the external part of the vesicle because their signals were found in the fractions of the density gradient where soluble proteins are expected. A similar observation was done recently where LGALS3BP is highly enriched in exomere nanoparticles (Zhang et al., 2018). Interestingly, a pool of fibronectin seems to be transported inside small vesicles as corroborated in our Western blot analysis and may reflect the overproduction of this protein in tumor sites (Libring et al., 2020; Park & Helfman, 2019).

In this work we developed a workflow to increase the efficiency of the traditional SEC method to study EV diversity. The Pre‐SEC workflow is a highly reproducible and scalable method suitable for mass spectrometry analysis. EV isolation by this workflow allows the elimination of impurities and increases the yield and purity of the Pre and SEC methods. Moreover, the Pre‐SEC strategy enables the fractionation of EV preparation while reducing the dependence on UC. This principle has implications for a clinical setting in which it is desirable to enrich EV‐associated proteins without time‐consuming protocols. The strategy presented here would be helpful for the analysis of EVs from different sources, including cell culture and body fluids, to have a better understanding of their heterogeneity and functional role.

## DISCLOSURE OF INTEREST

David A. Sinclair is a consultant to, inventor on patents owned or licensed to, board member and equity owner of EdenRoc companies (including MetroBiotech, ArcBio, Dovetail Genomics), Life Biosciences (including Iduna, Continuum, Sephagy), Cohbar, Alterity, Catalio Partners, TB12, InsideTracker, Immetas, NDLX, and Frontier Acquisition Corp. D.A.S. is an advisor to Zymo Research. Details and additional disclosures: https://sinclair.hms.harvard.edu/david‐sinclairs‐affiliations. Bogdan Budnik is scientific adviser to Merck & Co. All other authors report no conflict of interest.

## Supporting information

Supplementary information

Supplementary information

Supplementary information

Supplementary information

Supplementary information
