# Supplementary material for: Quantitative proteomic analysis of extracellular vesicle subgroups isolated by an optimized method combining polymer‐based precipitation and size exclusion chromatography
Source: J Extracell Vesicles. 2021 Apr 27;10(6):e12087. doi: 10.1002/jev2.12087 (PMC8077108; doi:10.1002/jev2.12087)
Supplement: Supplementary file 1 — Supplementary information [file JEV2-10-e12087-s002.pdf]

Supplementary Figures

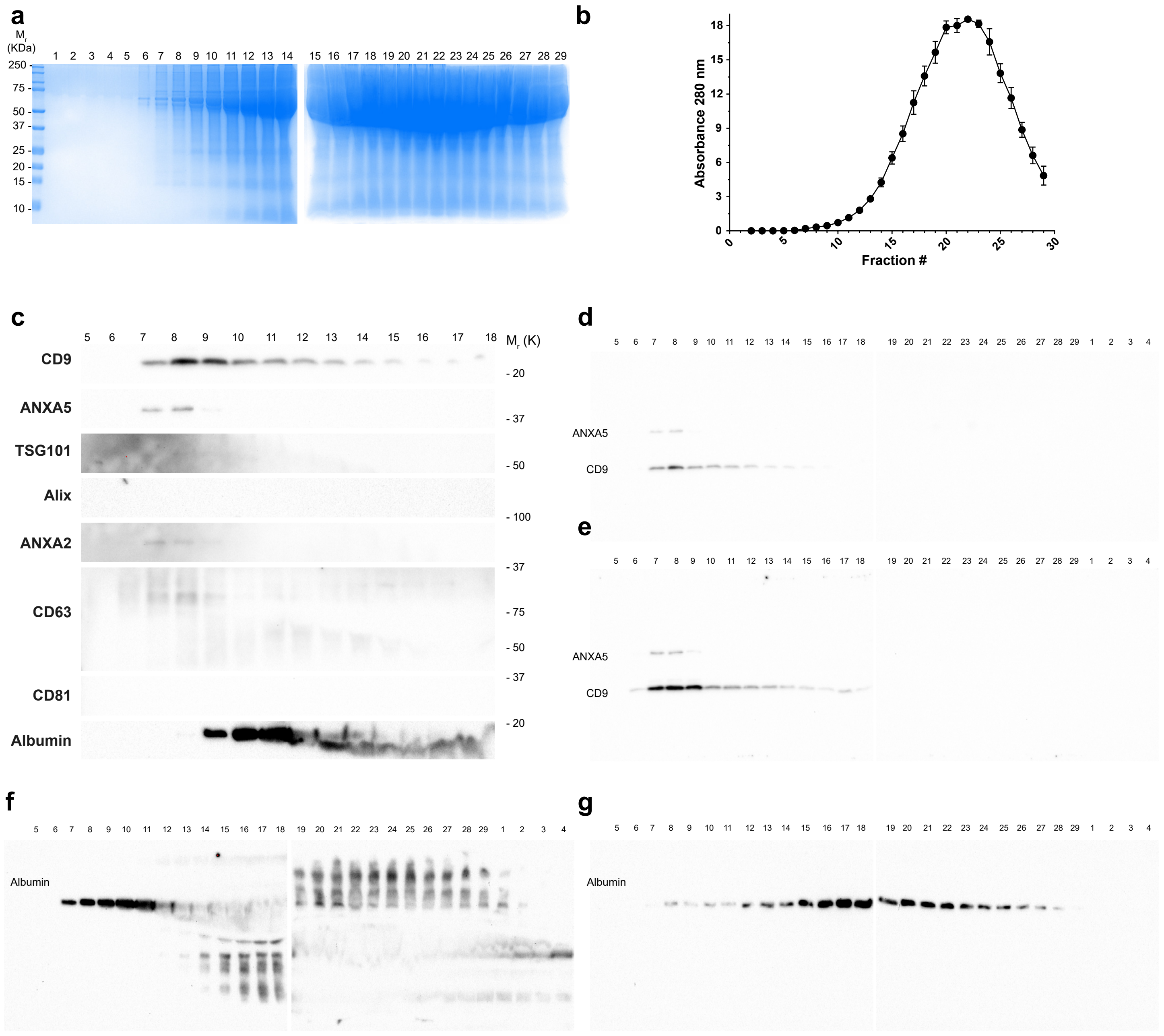

Supplementary figure 1. Supplementary figure 1. Characterization of MDA-MB-468 EVs isolated by the size exclusion chromatography (SEC) method. (a) Coomassie Blue staining of 29 fractions collected from SEC loaded in the gel at a constant volume (27μL/lane). (b) Relative protein content of the collected fractions using SEC. The highest value was obtained in fraction 22. (c) Distribution of EV protein markers from fractions 5 to 18 obtained by SEC loaded at a constant volume (27 μL/lane). Full image of representative Western blots showing CD9 and Annexin A5 signals in SEC (d) and Pre-SEC (e) fractions. Albumin signal in EV containing fractions from SEC (f) and Pre-SEC methods (g).

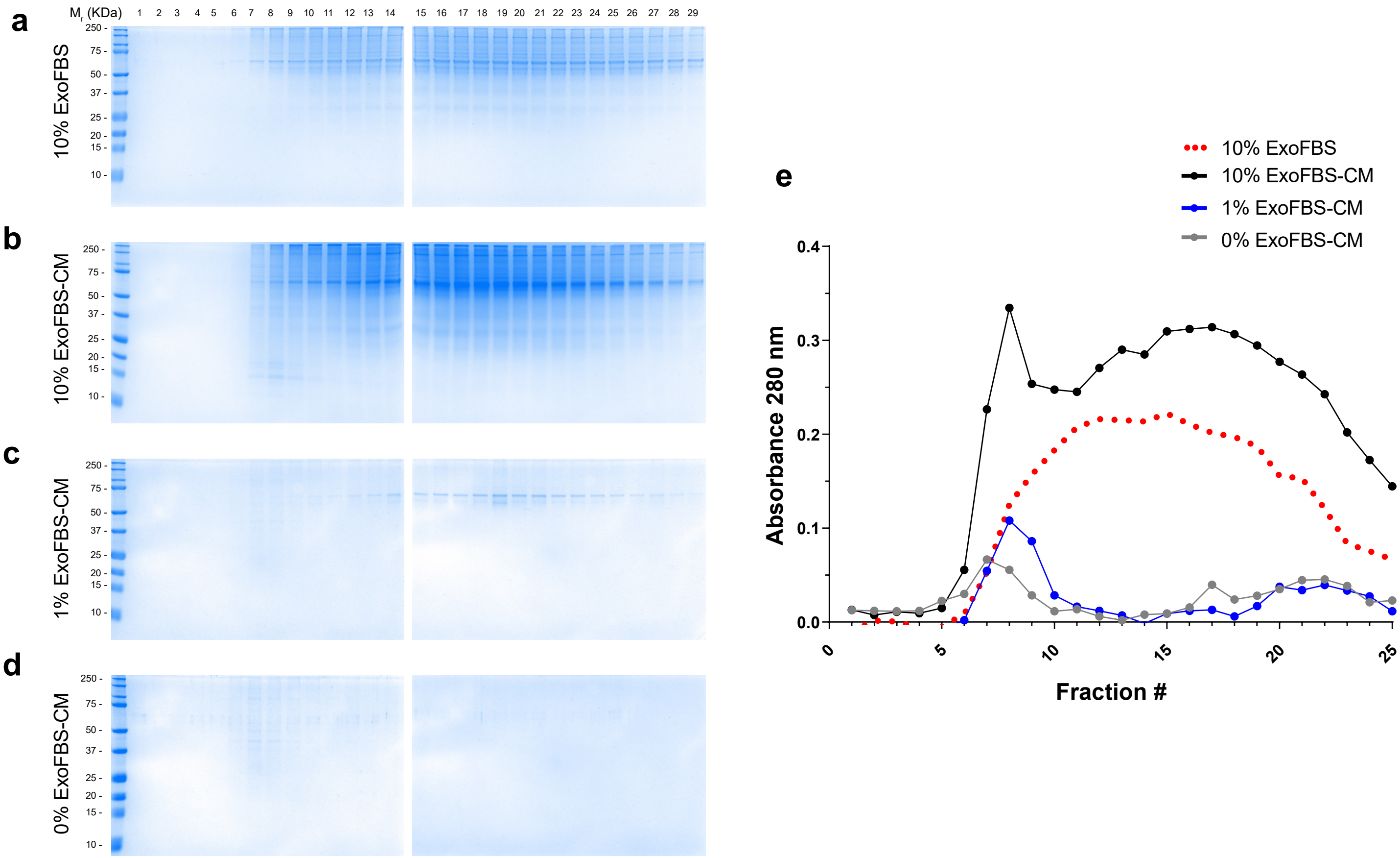

Supplementary figure 2. Effect of FBS concentration in protein recovery after Pre-SEC isolation. MDA-MB-468 cells were cultured with cell culture media supplemented with 10%, 1%, and 0% FBS. Conditioned media (CM) was collected after 48h of incubation and Pre-SEC method was performed. As a control, we performed the isolation procedure using cell culture media containing 10% EV-depleted FBS (10% ExoFBS). An equal volume of each fraction (27 µL/lane) was loaded into a Tricine-PAGE-SDS gel after Pre-SEC isolation. Coomassie Blue staining of the electrophoretic separation of Pre-SEC fractions obtained from 10% Exo-FBS (a), 10% ExoFBS-CM (b), 1% % ExoFBS-CM (c), and 0% ExoFBS-CM (d). (e) To determine the relative amount of protein in the first 25 fractions obtained from (a-d) we measured the absorbance at 280 nm. The decrease of ExoFBS concentration resulted in a diminishment of general protein content, but it remains a peak of protein in the region where EVs were expected.

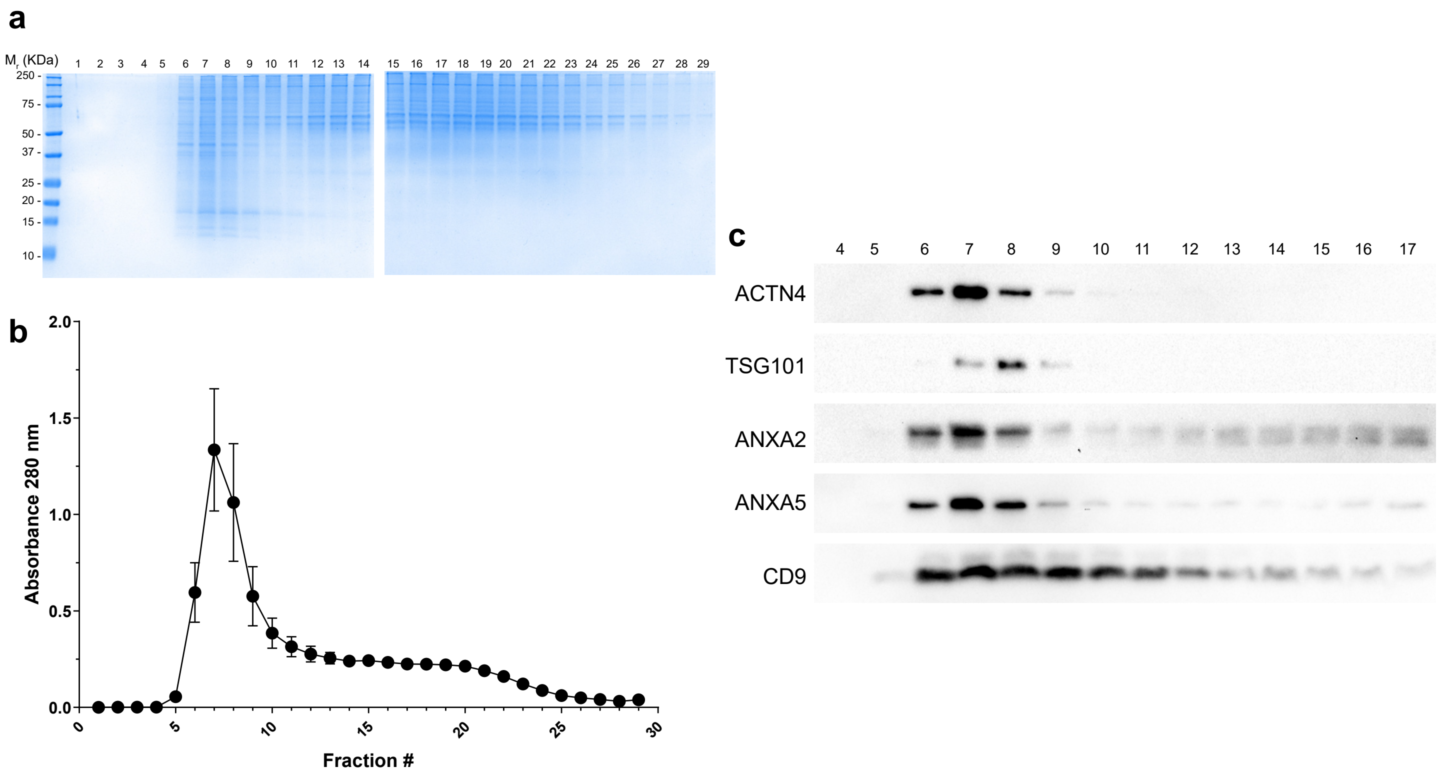

Supplementary figure 3. Analysis of EV markers in conditioned media supplemented with 1% ExoFBS. The conditioned media was collected from MDA-MB-468 cultures after 48 h of incubation. (a) Coomassie staining of electrophoresed proteins from Pre-SEC fractions. A constant volume (27 μL/lane) of the 29 fractions collected during EV isolation was loaded in each well. (b) Absorbance at 280nm of the 29 fractions collected during EV isolation using the Pre-SEC method (n=3). (c) Western blot at constant protein (20 μg/lane) of EV protein markers between fractions 4 to 17 after Pre-SEC isolation.

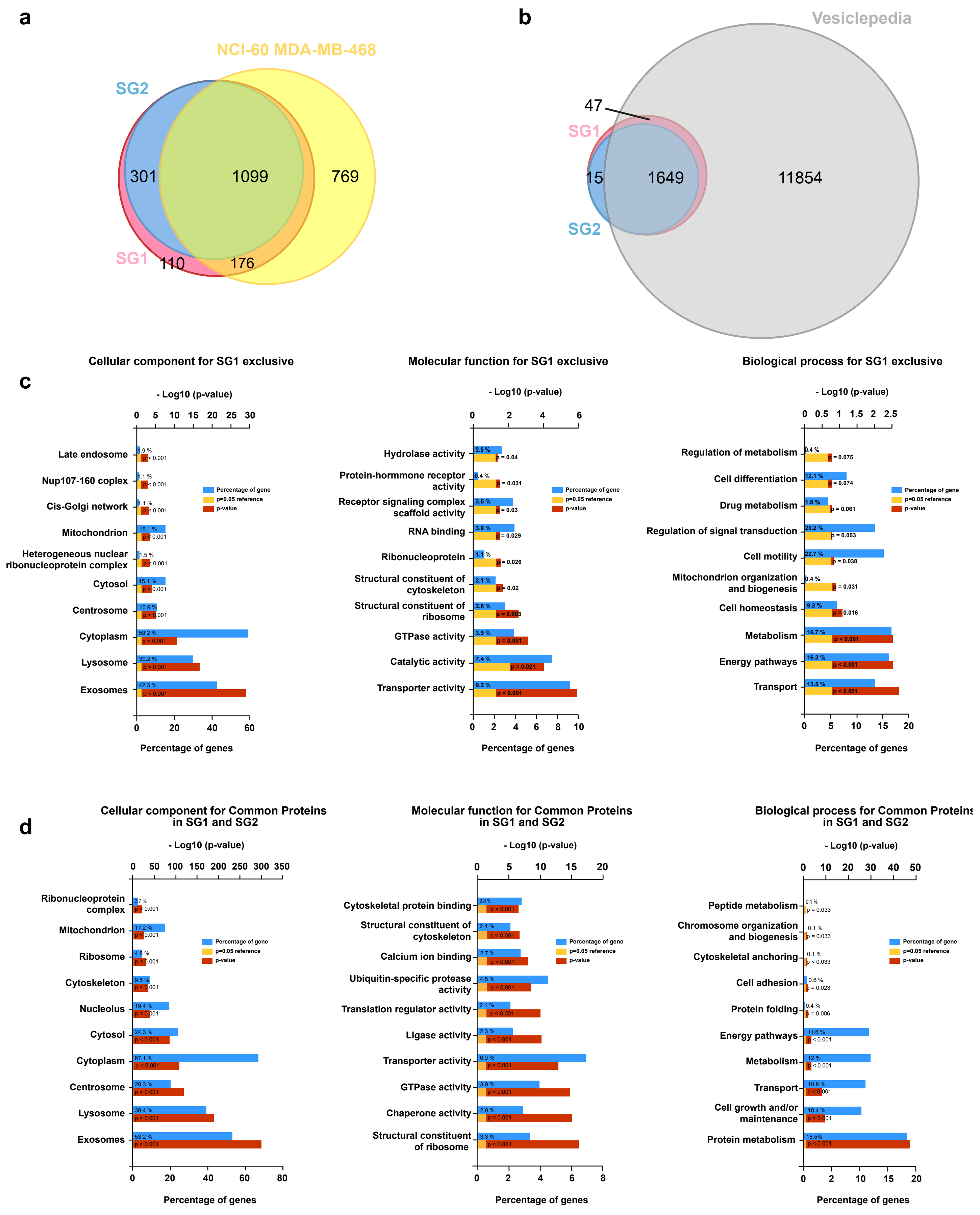

a
NCI-60 MDA-MB-468
SG2
SG1
301
1099
769
110
176
b
Vesiclepedia
47
SG1
SG2
15
1649
11854
c
Cellular component for SG1 exclusive
- Log10 (p-value)
Late endosome
Nup107-160 coplex
Cis-Golgi network
Mitochondrion
Heterogeneous nuclear ribonucleoprotein complex
Cytosol
Centrosome
Cytoplasm
Lysosome
Exosomes
Percentage of gene
p=0.05 reference
p-value
Percentage of genes
Molecular function for SG1 exclusive
- Log10 (p-value)
Hydrolase activity
Protein-hormmone receptor activity
Receptor signaling complex scaffold activity
RNA binding
Ribonucleoprotein
Structural constituent of cytoskeleton
Structural constituent of ribosome
GTPase activity
Catalytic activity
Transporter activity
Percentage of genes
Biological process for SG1 exclusive
- Log10 (p-value)
Regulation of metabolism
Cell differentiation
Drug metabolism
Regulation of signal transduction
Cell motility
Mitochondrion organization and biogenesis
Cell homeostasis
Metabolism
Energy pathways
Transport
Percentage of genes
d
Cellular component for Common Proteins in SG1 and SG2
- Log10 (p-value)
Ribonucleoprotein complex
Mitochondrion
Ribosome
Cytoskeleton
Nucleolus
Cytosol
Cytoplasm
Centrosome
Lysosome
Exosomes
Percentage of genes
Molecular function for Common Proteins in SG1 and SG2
- Log10 (p-value)
Cytoskeletal protein binding
Structural constituent of cytoskeleton
Calcium ion binding
Ubiquitin-specific protease activity
Translation regulator activity
Ligase activity
Transporter activity
GTPase activity
Chaperone activity
Structural constituent of ribosome
Percentage of genes
Biological process for Common Proteins in SG1 and SG2
- Log10 (p-value)
Peptide metabolism
Chromosome organization and biogenesis
Cytoskeletal anchoring
Cell adhesion
Protein folding
Energy pathways
Metabolism
Transport
Cell growth and/or maintenance
Protein metabolism
Percentage of genes

Supplementary figure 4. Proteomic and enrichment analysis of identified proteins in SG1 and SG2 vesicles derived from MDA-MB-468. Comparison of the number of proteins identified in SG1 and SG2 with the dataset of MDA-MB-468-derived EVs of Hurwitz et al., 2016 (a) and Vesiclepedia (b). Gene enrichment analysis for the 286 proteins found exclusively in SG1 associated with cellular component, molecular function and biological process GO terms (c). Gene enrichment analysis of shared proteins (1400) between SG1 and SG2 associated with cellular component, molecular function, and biological process GO terms (d).

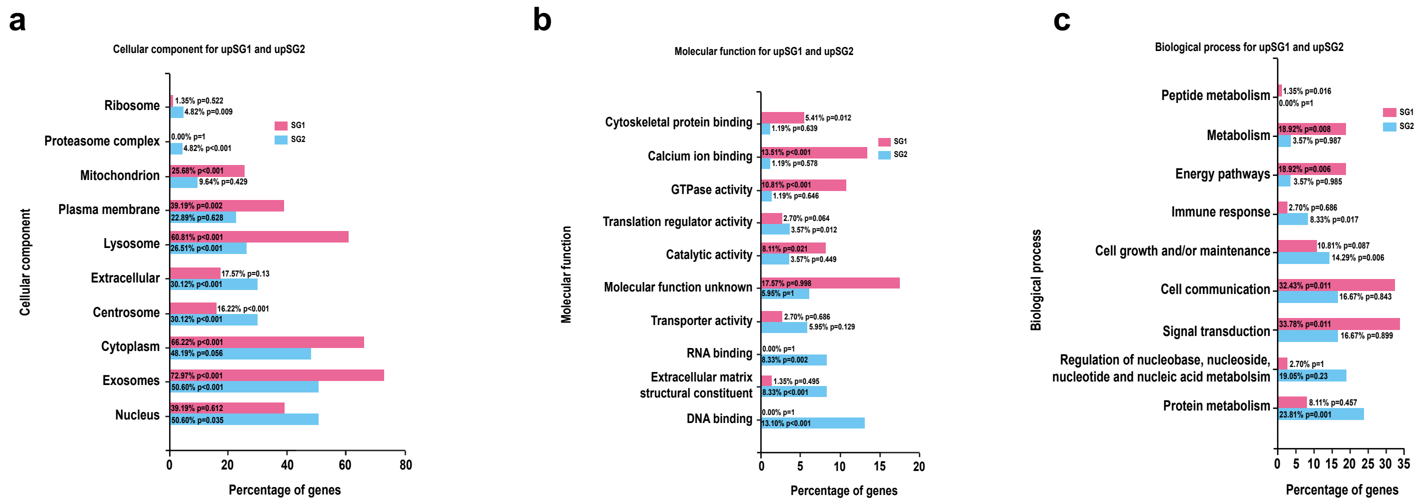

Supplementary figure 5. Gene enrichment analysis of upregulated proteins from SG1 (73 proteins) and SG2 (75 proteins). Protein enrichment was associated with cellular component (a), molecular function (b), and biological process (c) GO terms.

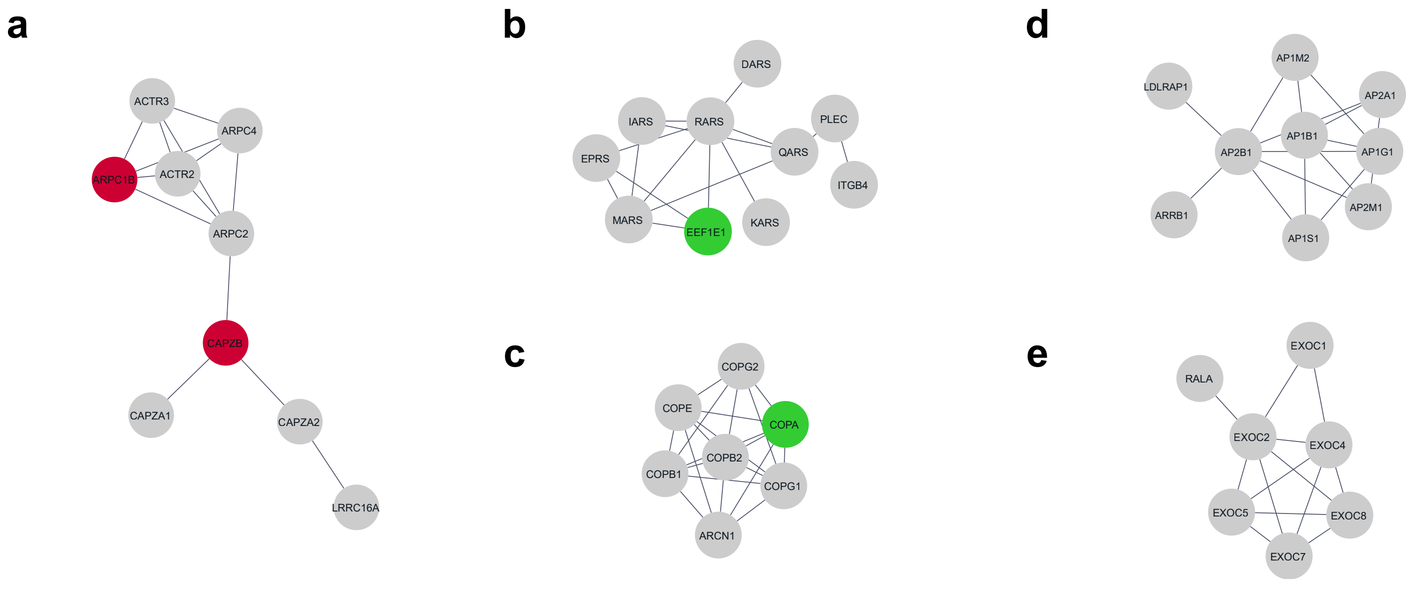

Supplementary figure 6. Protein-protein interaction networks of shared proteins by SG1 and SG2. In these subnetworks, the proteins enriched in SG1 are represented as red dots, and those enriched in SG2 as green dots. (a-d) Subnetworks containing functional modules associated with cytoskeleton reorganization (a), tRNA aminoacylation (b), non-clathrin-coated vesicles (c), clathrin-coated vesicles (d), and protein transport (exocyst) (e). The network analysis was performed with the STRING software (https://string-db.org). The network considers reported physical interactions of the 1400 proteins identified in both SG1 and SG2.

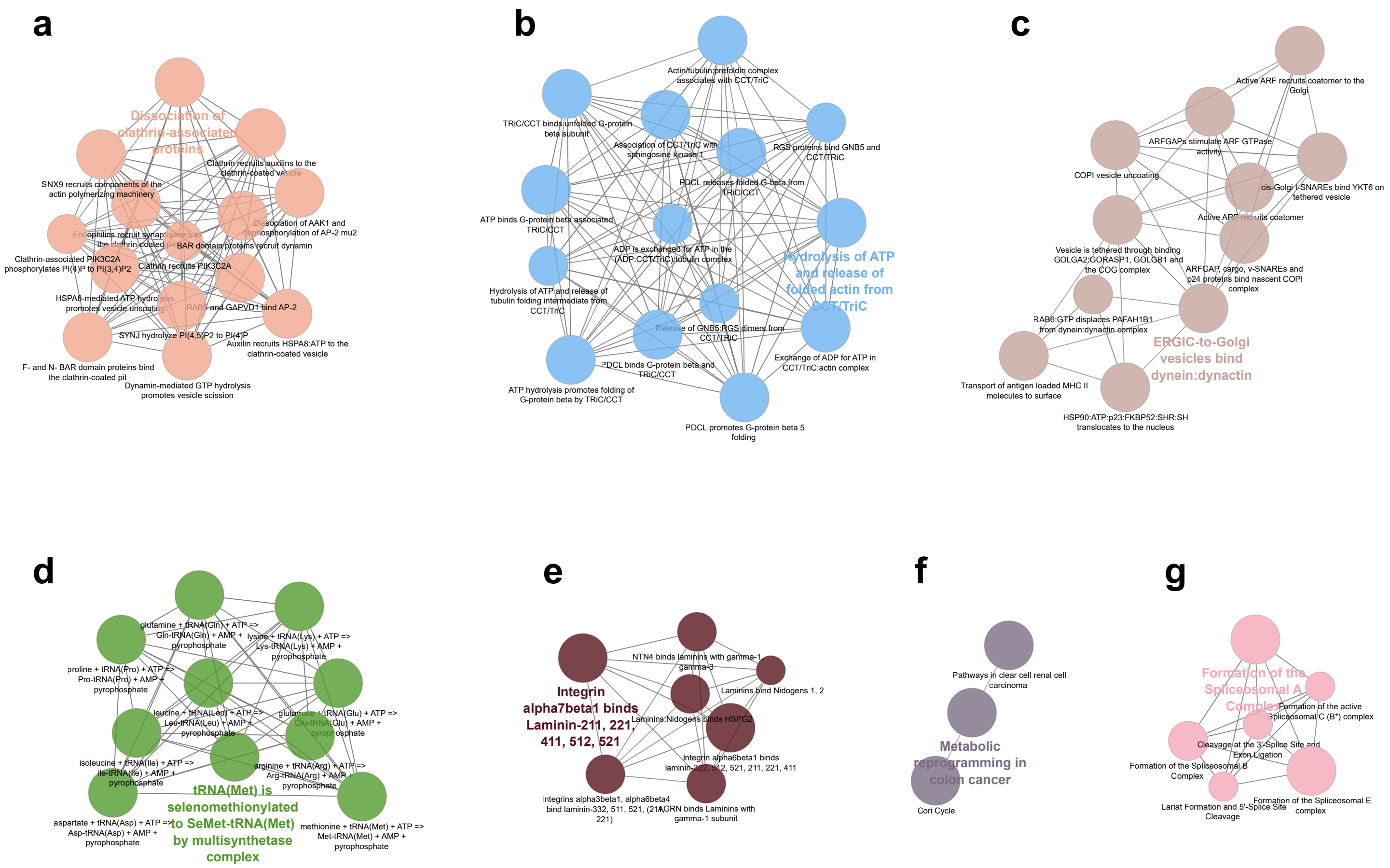

Supplementary figure 7. The enriched network of biological pathways in MDA-MB-468 EVs. Functional clustering was performed using ClueGo based on reactions in Reactome and Wikipathways considering 1400 proteins shared by SG1 and SG2. The most significant interactions are shown: (a) trafficking of clathrin-coated vesicles, (b) chaperonin function, (c) transport of COP I vesicles, (d) synthesis of tRNA, (e) extracellular matrix binding, (f) metabolic reprogramming and, (g) formation of the spliceosomal complex. Nodes represent the gene ontology (GO) terms, node color represents GO group, and node size represents statistical significance.
